# Enhanced Cellular Uptake and Transport of Bovine Lactoferrin Using Pectin- and Chitosan-Modified Solid Lipid Nanoparticles

**DOI:** 10.3390/pharmaceutics15082168

**Published:** 2023-08-21

**Authors:** Xudong Yao, Craig Bunt, Mengyang Liu, Siew-Young Quek, John Shaw, Jillian Cornish, Jingyuan Wen

**Affiliations:** 1 School of Pharmacy, Faculty of Medical and Health Science, The University of Auckland, Auckland 1142, New Zealandm.liu@auckland.ac.nz (M.L.); j.shaw@auckland.ac.nz (J.S.); 2 Department of Food Science, Otago University, Dunedin 9054, New Zealand; craig.bunt@otago.ac.nz; 3 Chemical Science, The University of Auckland, Auckland 1142, New Zealand; sy.quek@auckland.ac.nz; 4 School of Medicine, Faculty of Medical and Health Science, The University of Auckland, Auckland 1142, New Zealand

**Keywords:** cellular uptake, cellular transport, bovine lactoferrin, chitosan-modified solid lipid nanoparticles

## Abstract

Aim: The aim of this project is to use pectin- and chitosan-modified solid lipid nanoparticles for bovine lactoferrin to enhance its cellular uptake and transport. Methods: Solid lipid particles containing bovine lactoferrin (bLf) were formulated through the solvent evaporation technique, incorporating stearic acid along with either chitosan or pectin modification. bLf cellular uptake and transport were evaluated in vitro using the human adenocarcinoma cell line Caco-2 cell model. Results and Discussion: The bLf-loaded SLPs showed no significant effect on cytotoxicity and did not induce apoptosis within the eight-hour investigation. The use of confocal laser scanning microscopy confirmed that bLf follows the receptor-mediated endocytosis, whereas the primary mechanism for the cellular uptake of SLPs was endocytosis. The bLf-loaded SLPs had significantly more cellular uptake compared to bLf alone, and it was observed that this impact varied based on the time, temperature, and concentration. Verapamil and EDTA were determined to raise the apparent permeability coefficients (App) of bLf and bLf-loaded SLPs. Conclusion: This occurred because they hindered efflux by interacting with P-glycoproteins and had a penetration-enhancing influence. These findings propose the possibility of an additional absorption mechanism for SLPs, potentially involving active transportation facilitated by the P-glycoprotein transporter in Caco-2 cells. These results suggest that SLPs have the potential to be applied as effective carriers to improve the oral bioavailability of proteins and peptides.

## 1. Introduction

Lactoferrin (Lf) is an iron-binding glycoprotein of ~80,000 Dalton and consists of a single polypeptide chain of about 690 amino acids, first discovered from bovine milk in 1939 [1]. In recent years, there has also been considerable interest to develop Lf nutraceutical products for the improvement of general wellbeing, especially with respect to improving immune responses due to its antibacterial [2,3,4], antiviral [5,6], antitumor [7,8], anti-inflammatory [9,10], and anti-infection [11,12] properties. Moreover, the study carried out by Professor Cornish’s group has provided the first evidence that bLf exerts combined anabolic effects on osteoblasts and inhibitory effects on osteoporosis [13]. The current large-scale production techniques based on cation-exchange resins, followed by gel filtration, have been developed to isolate bLf from whey [14] and enable its use in products for the promotion of human health. However, the bioavailability of orally administered bLf is extremely low due to biochemical and physical barriers, including gastrointestinal proteases, the epithelial barrier of the small intestine, and efflux pumps [15]. Even after bLf is absorbed, another potential ‘barrier’ to consider is that of avoiding intracellular lysosome degradation in order to reach the blood supply. These obstacles contribute to the bioavailability levels of native bLf of less than 1% after oral administration [16,17].

Several approaches have been established to optimize oral delivery of bLf, and they can be broadly categorized into chemical and physical strategies [18]. The use of lipid-based delivery systems has been investigated for many years and is still a very promising concept. Dr. Alec Bangham invented liposomes in the 1960s [19], while the origin of solid lipid particles (SLPs) can be traced to lipid nanoparticles, introduced in the 1990s [20]. SLPs are often compared with liposomes due to the use of similar (lipid) materials for their construction. bLf has been shown to have improved pharmacological effects following oral administration by incorporation into liposomes [21,22]. It is unclear from the literature if bLf in a SLP formulation is a suitable delivery option.

In the past, we documented the creation of modified liposomes and solid lipid particles (SLPs) using hydrophilic polymers as carriers for delivering bLf [23]. However, liposomes have yet to be selected as the most ideal bLf carriers for the oral route. Upon exposure to a porcine bile extract and porcine pancreatin as a lipase source, liposome-based formulations showed rapid structural collapse and hydrolysis to high amounts of free fatty acid (FFA) [23]. Surface coating using either chitosan or pectin could not significantly delay and reduce the lipolysis of liposomes. While liposome-based formulations were subjected to long-term stability studies, similar results were obtained by exposure to high-temperature and high-humidity stress conditions. Such stress studies accelerated the thermodynamic motion of liposomes and promoted aggregation or disintegration, leading to leakage and the denature of encapsulated bLf [24]. In contrast, SLPs were able to maintain their particle morphology when exposed to simulated intestinal fluid (SIF), highly resist lipid digestion, as well as retain more bLf within the solid core, even under accelerated conditions.

**Figure 1:**
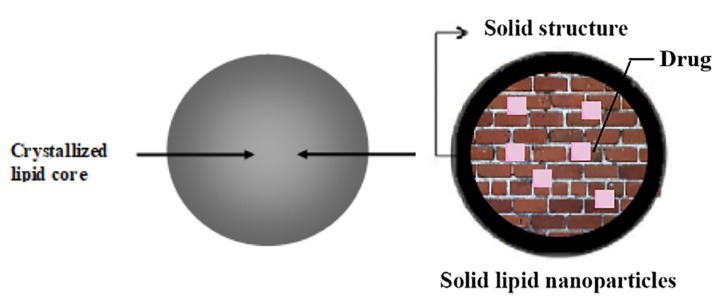
SLPs consist of a solid lipid or a mixture of solid lipids, which form an almost perfect crystalline structure.

SLPs possess two key features: they are colloidal carriers of submicron sizes, composed of lipids that are solid at body and room temperatures (Figure 1). Due to their diminutive particle dimensions, SLPs could potentially display bio-adhesive properties by becoming lodged within the inter-villar gaps of the gastrointestinal tract. This could extend their duration within the tract, resulting in improved bioavailability [25,26,27]. Optimized SLP formulations have been reported to maintain their physical integrity for at least three years [28,29,30]. Their physicochemical stability, low cost, and possibility for large-scale production, compared to liposomes, make SLPs highly suitable for further research as a delivery vehicle for oral delivery of proteins and peptides [31,32]. However, the safety profile and the mechanisms involved in the cellular uptake and transport of polymer-modified SLPs as vehicles for drug delivery have not undergone assessment.

The Caco-2 cell line, derived from human adenocarcinoma, has been established as a representation of the intestinal epithelium [33,34,35]. Fogh et al. initially isolated Caco-2 cells from a human colon adenocarcinoma [36]. The Caco-2 cell system serves as an in vitro model for intestinal absorption and is commonly employed by both the industry and academia for assessing intestinal permeability. These cells also exhibit the presence of nutrient and drug transporters, enabling investigations into mechanisms related to carrier-mediated uptake and efflux [37]. Our objective was to investigate the interactions between pectin- or chitosan-modified SLPs and biological membranes using a Caco-2 cell monolayer model. An initial assessment of the cytotoxicity was conducted using the MTT assay to evaluate the safety of SLPs as a viable oral delivery approach. Subsequently, investigations into cellular uptake were undertaken to quantify the delivery capability of the SLP formulations. Finally, various absorption inhibitors and enhancers were applied to investigate the transport properties of SLPs that might be affected by the drug intestinal cellular efflux transporters.

## 2. Materials and Methods

### 2.1. Materials

Bovine lactoferrin (bLf), derived from bovine colostrum, was provided as a generous donation by Fonterra, located in Palmerston North, New Zealand. Stearic acid (Grade I, with a purity of at least 99% according to gas chromatography) and a combination of high- and low-methoxyl pectin sourced from apples were used, as well as 3-[4,5-dimethylthiazol-2-yl]-2,5 diphenyltetrazolium bromide (MTT), fluorescein isothiocyanate (FITC), fluorescein sodium salt, sodium azide, verapamil, MK-571 sodium salt hydrate, and ethylenediaminetetraacetic acid (EDTA), acquired from Sigma Aldrich, based in St. Louis, MO, USA. Soybean lecithin was obtained from BDH, located in Poole, UK. Poloxamer 188 was procured from BASF in Ludwigshafen, Germany. Chitosan with low viscosity was purchased from Fluka, situated in Park Rabin Rehovot, Israel. Dulbecco’s Modified Eagles’ Medium (DMEM), fetal calf serum, penicillin-streptomycin-glutamine, nonessential amino acids, trypsin-EDTA, sterile phosphate-buffered saline (PBS) (pH 7.4), and Hank’s balanced salt solution (HBSS) buffer (pH 7.4) were purchased from Life Technologies (Carlsbad, CA, USA). The 4′6-diamindino-2-phenylindole (DAPI) was purchased from Invitrogen (Auckland, New Zealand). The Caco-2 cell line was purchased from the American Type Culture Collection (Manassas, VA, USA). All other reagents and chemicals were of analytical grade.

### 2.2. Analysis of Bovine Lactoferrin

The quantification of the lactoferrin concentration in the microgram range was accomplished using an RP-HPLC technique described in a prior study [38]. In essence, substances were separated on a C18 HPLC column (Jupiter 5u C18 300R, 250 × 4.6 mm, 5 mm, Pheonomenex, Auckland, New Zealand), equipped with a security C18 cartridge (10 × 3.0 mm). Mobile phase A consisted of 0.1% TFA in water and acetonitrile (95:5 *v*/*v*), while mobile phase B consisted of 0.1% TFA in water and acetonitrile (5:95 *v*/*v*). The mobile phases were filtered through a 0.2 µm nylon membrane and degassed prior to use. A constant flow rate of 0.5 mL/min was used. The injection volume of the analyzed samples was 50 µL, and the column temperature was maintained at 37 °C. Absorbance was measured at a wavelength of 210 nm. The elution process commenced with an isocratic elution using 35% mobile phase B for 1 min, followed by a gradual increase to 60% mobile phase B over 19 min. A subsequent 5-min period (post-time) was allocated for equilibration.

The lactoferrin concentration, in the ng range, was evaluated by the ELISA assay. The creation of a standard curve for bLf was accomplished through the employment of the bLf ELISA Quantitation Set Protocol from Bethyl, located in the USA. In summary, a stock solution of bLf at a concentration of 1 mg/mL was diluted using the provided sample diluent, resulting in a progression of standards spanning concentrations of 7.8, 15.6, 31.25, 62.5, 125, 250, 500, and 1000 ng/mL. The assay was conducted using a 96-well plate included in the kit. The ELISA kit was applied according to the manuals supplied by the manufacturer, as follows: The affinity purified antibody was diluted with a coating buffer (1:100). Then, 100 µL of the diluted antibody was added into each well and incubated for 60 min at room temperature. After aspirating the antibody solution from each well, the plates were washed 3 times using 300 µL of wash buffer. Potential free binding sites of the wells were blocked with post-coat solution (200 µL). Following a 30-min incubation at ambient temperature, the plate underwent an additional round of 3 washes using the washing buffer. Subsequently, a set of standards (100 µL each) was dispensed into their designated wells in triplicate and allowed to incubate for 60 min at room temperature. After an additional 5 rounds of washing with 300 µL of wash buffer, 100 µL of a diluted solution of the goat anti-bLf-HRP conjugate detection antibody was introduced into each well and incubated for 60 min. After the removal of the HRP detection antibody, the plate was washed 3 times using 300 µL of wash buffer. Equivalent volumes of substrate reagents A and B (comprising TMB peroxide) were combined, and 100 µL of the mixture was introduced into every well, followed by a 15-min incubation period. The plate was covered with aluminum foil, facilitating the development of the enzymatic color reaction in a dark environment. Finally, stop solution (100 µL of 2 M H_2_SO_4_) was added into the wells, and absorbance was measured at 450 nm using a SepctraMax Plus 384 (Molecular Devices, San Jose, CA, USA), with a 20 min addition of acid. The analysis of the data was conducted utilizing MasterPlex 2010 software version 2.0.0.68.

### 2.3. Analysis of Total Proteins in Caco-2 Cells

The complete protein content within the cells was assessed using the Pierce^®^ bicinchoninic acid (BCA) protein assay from Thermo Scientific, Waltham, MA, USA. The assay was applied according to the manuals supplied by the manufacturer, as follows: A stock solution of bovine serum albumin (BSA) (2 mg/mL) was diluted to yield a series of 25, 125, 250, 500, 750, 1000, 1500, and 2000 µg/mL standards. Then, 25 µL of each standard BSA or sample was transferred in triplicate into a 96-well plate, followed by adding 200 µL of the BCA working reagent (WR) (prepared by mixing 50 parts of BCA Reagent A with 1 part of Reagent B). The plate was placed on a plate shaker for 0.5 min and incubated at 37 °C for 30 min. Subsequently, the plate was allowed to return to ambient temperature, and the absorbance was measured using a SpectraMax Plus 384 spectrophotometer from Molecular Devices, USA, at a wavelength of 562 nm.

### 2.4. Preparation and Characterization of Unmodified, Pectin-, and Chitosan-Modified SLPs

The physicochemical properties of SLPs, including the sustained release profile, drug–lipid–polymer interactions, and stability against enzymatic degradation, have been investigated in a previously reported study [23]. The particle sizes of the optimized SLPs reported in that study were used for this study. In brief, the particle size, size distribution, and surface charge of SLPs were measured by a NanoSight NS300 (Malvern Instruments Ltd., Malvern, UK) and a Zeta Sizer (Malvern Instruments Ltd., UK) and analyzed by NanoSight Software 2.0 at 25 °C, with 5-time dilution by phosphate-buffered saline (PBS). SLP dispersions were prepared by the emulsion/solvent evaporation method, as follows. Stearic acid (28 mg) and 12 mg of lecithin were completely dissolved in 10 mL of the organic phase containing acetone and dichloromethane (DCM) (1:4 *v*/*v*). Subsequently, the aqueous phase was generated by dissolving 3 mg of bLf in 5 mL of phosphate-buffered saline (PBS) with a pH of 7.4. This solution was gradually introduced into the organic phase within a bath sonicator. The blend was sonicated using a probe sonicator for 1 min at a frequency of 0.5 cycles and an amplitude of 50%, all conducted in an ice bath. The resulting water-in-oil (w/o) primary emulsion was promptly poured onto the second aqueous phase. The second aqueous phase was prepared by dissolving 10 mg of poloxamer 188 in 25 mL of PBS with a pH of 7.4. The resulting water-in-oil-water (w/o/w) emulsion was continuously stirred at 30× *g* to facilitate solvent evaporation at room temperature for approximately 6 h. The suspension of solid lipid particles (SLPs) was subsequently subjected to centrifugation at 75,000× *g* for 1 h, at 4 °C, to separate unencapsulated bLf from bLf-loaded SLPs. The unconstrained bLf in the supernatant was analyzed via RP-HPLC to calculate the entrapment efficiency (EE) using the formula: % EE = (Wt − Wf)/Wt × 100%, where Wt represented the initial bLf quantity and Wf indicated the amount of unencapsulated bLf in the supernatant. For the coating of SLPs, uncoated SLPs were dispersed into 30 mL of PBS (0.1 M, pH 7.4), and subsequently, 20 mg of either pectin or chitosan was introduced at a polymer-to-solid lipid mass ratio of 1:2. The mixtures were left to interact overnight to allow the polymer to adhere to the particles. After an additional centrifugation step at 75,000× *g* for 1 h, at 4 °C, the sediment of pectin- or chitosan-coated SLPs was collected and subjected to freeze-drying (Labconco, Kansas City, MO, USA) for dehydration. The dried samples were stored in a desiccator at room temperature until they were analyzed.

### 2.5. Stability of bLf in Cell Culture Media (DMEM and HBSS at pH 7.4)

To identify the possibility of bLF degradation in the culture medium, bovine lactoferrin (100 µg/mL) was dissolved in DMEM (pH 7.4) or HBSS (pH 7.4) and incubated at 37 °C for 6 h. Then, 100 µL samples were withdrawn at specified time intervals. Each sample was examined by the RP-HPLC method to identity the integrity of the bLf peak retention time.

### 2.6. In Vitro Cell Line Studies

#### 2.6.1. Cell Culture

Caco-2 cells were cultured in a complete DMEM medium (with a pH of 7.4), enriched with 10% fetal calf serum, 1% nonessential amino acids, and 1% penicillin-streptomycin-glutamine solution (at concentrations of 100 U/mL, 100 µg/mL, and 2 mM, respectively). The cells were routinely cultivated in T-75 tissue culture flasks within a humidified environment containing 5% CO_2_ and 95% air, maintaining a temperature of 37 °C. The medium was exchanged for fresh complete DMEM medium every 3 days until cells grew to 90% confluence. Viable cells were determined via the trypan blue exclusion method based on the fact that trypan blue dye can diffuse through the cell membrane of dead cells only. For sub-culturing, the cells were dissociated with 0.25% trypsin–EDTA, split in a ratio of 1:3.

#### 2.6.2. Cytotoxicity Studies

The cytotoxicity of bLf and bLf-loaded unmodified, chitosan-, and pectin-modified SLPs towards Caco-2 cells was examined using the MTT assay for the assessment of cell viability [39,40]. Briefly, 10^4^ cells/cm^2^ per well were sub-cultured into 96-well plates. After the monolayer of cells was formed for 24 h at 37 °C, the medium was replaced with serum-free medium containing various concentrations of bLf, bLf-loaded unmodified, chitosan-, and pectin-modified SLPs (12.5–300 µg/mL in serum-free medium), respectively, while cells treated with serum-free medium were used as a control. After the 4 h, 8 h, and 12 h exposure, 100 µL of MTT (0.5 mg/mL in the serum-free medium) was added to each well, and then the cells were incubated for another 2 h at 37 °C. The liquid above the cell sediments was removed, and the resulting formazan deposits were dissolved by introducing 100 µL of a solution containing 0.04 M HCl in isopropyl alcohol. This mixture was subjected to shaking on a plate for a duration of 15 min. The assessment of cell viability was accomplished through the MTT assay, which gauged the enzymatic conversion of yellow tetrazolium MTT to a purple formazan compound. The measurement occurred at a wavelength of 570 nm using a SpectraMax Plus 384 spectrophotometer from Molecular Devices, USA [39,40]. The percentage of cellular activity was computed using the formula: cell activity (%) = (Aexp − Aneg)/(Acon − Aneg) × 100%, where Aexp represents the absorbance value of the experimental group at 570 nm, Aneg signifies the absorbance value of the blank group at 690 nm, and Acon corresponds to the absorbance value of the control group at 570 nm. The cell viability of the specimen was denoted by its IC_50_ value, which stands for the concentration in milligrams of the substance per milliliter that reduces the cell viability by 50%. Concentrations falling within a safe range were employed for subsequent transport experiments.

#### 2.6.3. Confocal Microscopy Analysis

First, bLf was labeled with fluorescein isothiocyanate (FITC) [41]. In brief, a solution was prepared by dissolving 50 mg of bLf and 5 mg of FITC in 5 mL of 0.1 M carbonate buffer at a pH of 8.5. The mixture was then stirred in darkness for a duration of 8 h at a temperature of 4 °C. The mixtures were fractionated by gel permeation chromatography using a Sephadex G-25M column (Sigma Aldrich, USA). In brief, 1 mL of the mixture was loaded on to the top of the column gel bed and eluted through the column with 10 mL of PBS (0.01, pH 7.4). Then, 1 mL of the fractions was collected, and the absorbance of each fraction was measured at 280 nm. Two bands were visible during elution. The conjugates were present in the first band (fractions 3–5, absorbance > 0.4). Fractionated FITC-bLf conjugates were dehydrated by a freeze-dryer (Labconco, USA), and encapsulated by the same preparation method as the unlabeled bLf formulation, as described above.

Caco-2 cells were placed into two-well chamber slides (BD Falcon, Miami, FL, USA) at a density of 104 cells per square centimeter and cultured using complete DMEM culture medium. On the second day (cultured for 24 h), cellular monolayers were subjected to a preliminary incubation with 1 mL of HBSS (Hank’s Balanced Salt Solution) for a duration of 15 min at a temperature of 37 °C. After equilibrating, the medium was replaced with 1 mL suspensions of FITC-bLf, FITC-bLf-loaded unmodified, chitosan-, and pectin-modified SLPs (10 µg/mL in HBSS), followed by incubation for 1 h at 37 °C [42]. Subsequently, the cells underwent eight rounds of cold sterile PBS (pH 7.4) washing. This was followed by fixation using freshly prepared 4% p-formaldehyde (PFA, pH 7.4) for a duration of 20 min. After fixation, the cell nuclei were stained with DAPI (at a concentration of 100 nm in PBS) for 10 min. Once the culture chambers were removed, the slides were extensively washed with PBS and then mounted with CITI-Fluor, a medium designed to reduce photobleaching during microscopy observations. To secure the slides, cover slips were positioned on top and sealed using nail polish. In preparation for the experiment, the slides were stored at 4 °C while being shielded from light. Slides were observed by the laser scanning confocal microscope (Leica TCS SP2, Bio-strategy, Rosedale, New Zealand). Samples were excited with 488 nm (green) and 405 nm (blue) laser lines. The fluorescence images were analyzed using Leica Confocal Software (LCS) version 2.61 (Bio-strategy, Rosedale, New Zealand).

#### 2.6.4. Quantitative Analysis of the Uptake of bLf and bLf-Loaded SLPs

**Figure 2:**
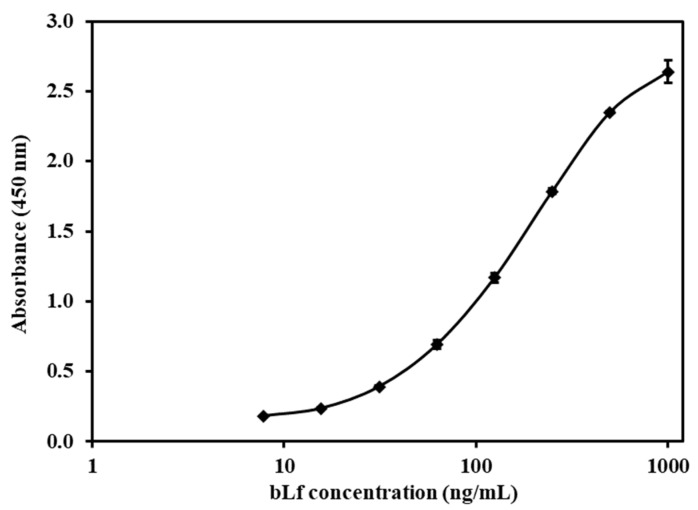
ELISA standard curve of bLf, ranging from 7.8 to 1000 ng/mL. y = ((A − D)/(1 + (x/C) B)) + D. A: 0.141, B: 1.307, C: 187.368, D: 2.934, R^2^: 0.999. Each point represents the mean ± SD (*n* = 3).

**Figure 3:**
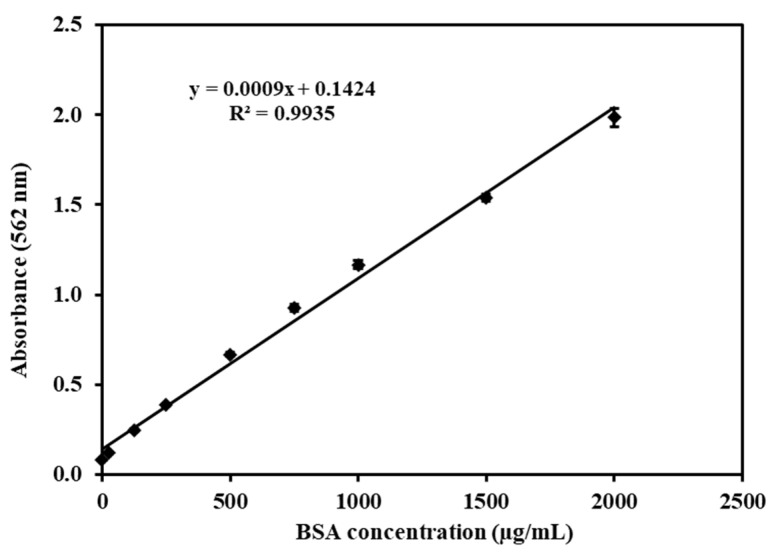
BCA standard curve of total cell proteins, ranging from 0 to 2000 µg/mL. Each point represents the mean ± SD (*n* = 3).

A total of 5 mL of a Caco-2 cell suspension containing 10^5^ cells/cm^2^ was introduced onto 60 mm plastic dishes (Corning, NY, USA). These cultures were supplied with complete DMEM every 3 days and maintained in an incubation environment at 37 °C, with a composition of 5% CO_2_ and 95% relative humidity. Upon reaching a confluence level of 90%, the culture medium was substituted with 2 mL of HBSS (Hank’s Balanced Salt Solution). After a 30-min incubation at 37 °C, the medium was substituted again, this time with 1 mL suspensions containing bLf, bLf-loaded non-modified, chitosan-modified, and pectin-modified SLPs (at concentrations of 25, 50, and 100 µg/mL in HBSS). These suspensions were then incubated for a period of 4 h, both at 4 °C and 37 °C, respectively. This was carried out to investigate the influence of the incubation temperature and drug concentration on particle uptake. For time-dependent uptake experiments, the medium was replaced with 1 mL of 100 µg/mL suspensions of free bLf and bLf-loaded SLPs in HBSS per well, and the plate was incubated for 30 min, 1 h, 2 h, and 4 h at 37 °C, respectively. Then, the cells were washed with cold sterile PBS (pH 7.4) five times and solubilized in 1 mL of 10% Triton X-100 in methanol, followed by extraction of bLf. A volume of 25 µL of cell lysates extracted from each well was utilized for the BCA protein assay (Thermo Scientific, USA) to quantify the cellular protein content. The remaining portion of the cell lysates underwent ELISA analysis (Bethyl, Montgomery, TX, USA) to assess the bLf quantity. The bLf uptake by Caco-2 cells was computed utilizing the standard curves (Figure 2 and Figure 3) and presented as the amount of drug (in nanograms) taken up per microgram of cellular protein.

#### 2.6.5. Transepithelial Transport of bLf and bLf-Loaded SLPs

Caco-2 cells were introduced onto Transwell^®^ inserts (with a pore diameter of 0.4 µm and an area of 1.13 cm^2^, manufactured by Corning, Corning, NY, USA) at a density of 10^5^ cells per square centimeter. These cells were cultivated within a humid environment composed of 5% CO_2_ and 95% air, maintaining a temperature of 37 °C. The complete DMEM medium was refreshed every 3 days, with 0.5 mL of the medium on the apical (AP) side and 1.5 mL on the basolateral (BL) side of the inserts. The integrity of the cellular monolayer was evaluated by observing the transepithelial electrical resistance (TEER) [43].

TEER was assessed utilizing a Millicell-ERS Volt-Ohm meter combined with a set of chopstick electrodes (manufactured by Millipore Corp, Billerica, MA, USA). To sterilize the electrodes, they were exposed to 70% ethanol for 15 min and then air-dried for 15 s. Subsequently, the shorter electrode was submerged in the media on the AP side, while the longer electrode was placed in the media on the BL side of the cell culture insert. Care was taken that the electrodes did not touch the cell monolayer. The TEER measurements were performed at 3-day intervals until the TEER values exceeded 350 Ω·cm^2^ (after ~24 days post-seeding in this study). TEER (Ω·cm^2^) = (*R*_cells_ − *R*_blank_) × *A*, where *R*_cells_*, R*_blank_, and *A* are the measured TEER value, the TEER value of the blank transwell, and the membrane surface area of the transwell (1.13 cm^2^), respectively. Caco-2 cell monolayers displaying TEER measurements surpassing 350 Ω·cm^2^ were deemed to signify a fully developed and dense Caco-2 cell monolayer, suitable for the transport studies.

Prior to the transport experiments, Caco-2 cell monolayers were equilibrated with HBSS for 30 min and removed by aspiration. All transport studies were conducted at 37 °C. Then, 0.5 mL suspensions of bLf, bLf-loaded unmodified, chitosan-, and pectin-modified SLPs (100 µg/mL in HBSS) were added on the AP side, while the BL side of the inserts contained 1.5 mL of HBSS. At 0, 60, 120, 240, and 360 min of incubation, 200 µL was withdrawn from the BL receiving chamber and was immediately replenished with an equal volume of pre-warmed HBSS (37 °C). The concentration of bLf in the transport medium was analyzed by ELISA, as described above. Additionally, the impact of substances that either hindered or enhanced absorption on transport was examined. In this regard, an adenosine triphosphate (ATP) inhibitor (sodium azide at a concentration of 10 mM), a permeability glycoprotein (P-gp) inhibitor (verapamil at a concentration of 100 µM), a multidrug resistance-associated protein 2 (MRP2) inhibitor (MK-571 at a concentration of 100 µM), and an absorption enhancer (EDTA at a concentration of 10 mM) were introduced to the AP side of the experiment. These compounds were combined with 0.5 mL suspensions of bLf and bLf-loaded SLPs at a concentration of 100 µg/mL, respectively. All inhibitors were dissolved in DMSO and then diluted using HBSS. The transport attributes of bLf and bLf-loaded SLPs across Caco-2 cell monolayers were represented in terms of the transport rate. This transport rate, denoted as flux, was quantified in μg/min^−1^/cm^−2^ and determined using the formula: flux = (dM/dt)/A, where dM/dt signifies the cumulative quantity of bLf traversing to the basolateral (BL) side per unit of time (µg/min), and A stands for the surface area of the membrane within the insert (1.13 cm^2^). The P_app_ was expressed in cm/s and was calculated by: P_app_ = (dM/dt)/(60 × A × C), where C is the initial concentration (100 µg/mL). The TEER values of the monolayers were measured at 1 h intervals during the experimental period, ensuring the integrity of the monolayers.

### 2.7. Statistical Analysis

All results were presented as mean values accompanied by their corresponding standard deviations (SD). The IC_50_ value was determined through nonlinear regression analysis utilizing GraphPad Prism Version 5.02 software (GraphPad Software Inc., San Diego, CA, USA). To evaluate the statistical significance of variations among the data, a one-way analysis of variance (ANOVA) was conducted using SPSS Version 22 software (Chicago, IL, USA). Differences were deemed statistically significant at a significance level of *p* < 0.05.

## 3. Results and Discussion

### 3.1. Analysis of bLf and SLP Preparation

The samples of bLf were subjected to triplicate testing employing the aforementioned ELISA technique, and the respective bLf concentrations in the samples were determined using the equation depicted in Figure 2. The quantification of total protein within each sample was accomplished by comparing it to the BSA standard curve illustrated in Figure 3.

As shown in Table 1, the particle size of SLPs was 283.1 ± 4.4 nm, and the sizes of those modified by chitosan and pectin were 518.5 ± 18.9 and 459.5 ± 22.2 nm, respectively. An around −10 mV surface charge of SLPs provides adequate charge repulsion amongst nanocarriers to maintain their physical stability for storage. The bLf encapsulation efficiency was approximately 92.10% within the SLPs, which equated to around 2.7 mg of bLf loaded in 40 mg of total lipids.

### 3.2. Stability of bLf in HBSS and DMEM

**Figure 4:**
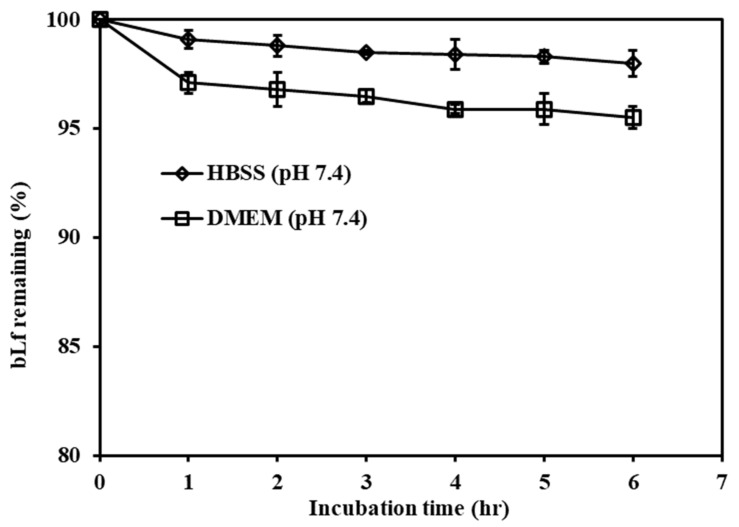
Stability profiles of 100 µg/mL of bLf in HBSS buffer (pH 7.4) and DMEM (pH 7.4) during 6 h of incubation at 37 °C. Each point represents the mean ± SD (*n* = 3).

The bLf (100 µg/mL) was incubated in both HBSS and DMEM at 37 °C, as shown in Figure 4, and 98.0 ± 0.6% and 95.5 ± 0.5% of bLf still remained after 6 h of incubation in the HBSS and DMEM, respectively. Therefore, the degradation of bLf during the experiment process was neglected.

### 3.3. In Vitro Cytotoxicity

**Figure 5:**
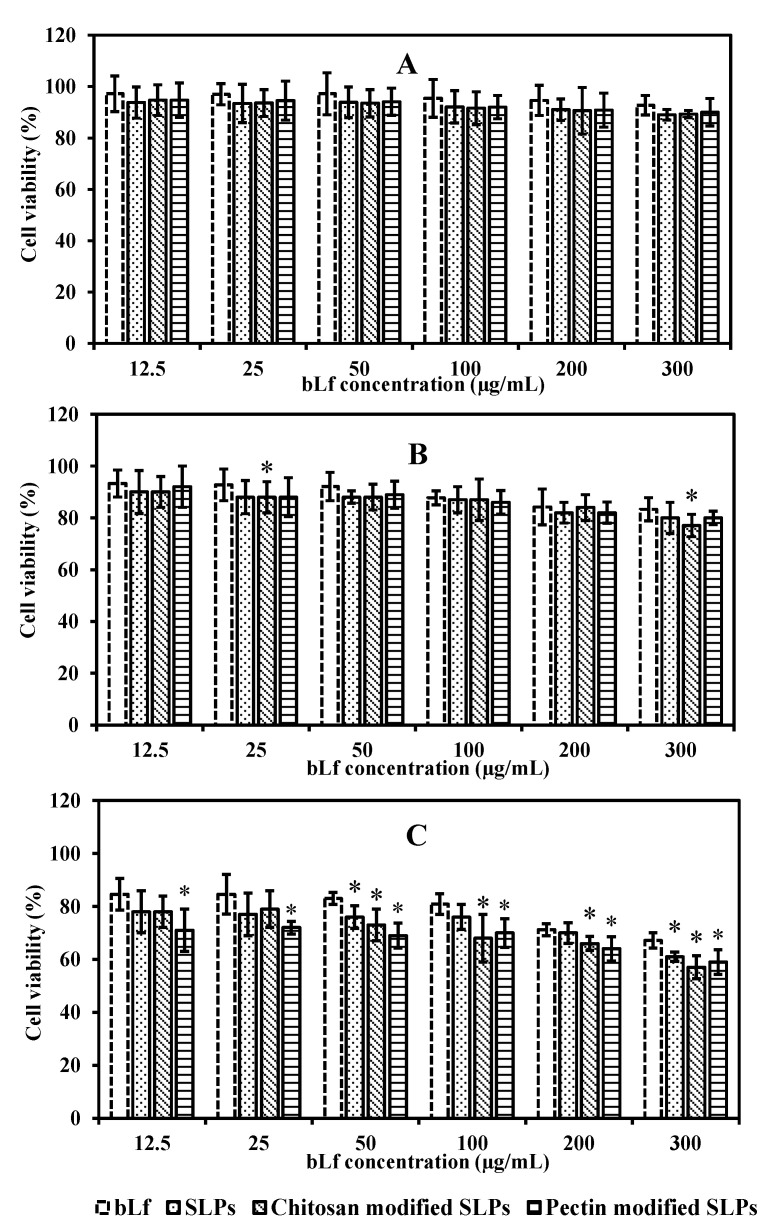
Cytotoxicity assay of bLf, bLf-loaded unmodified, chitosan-, and pectin-modified SLPs on Caco-2 cells at 4 h (**A**), 8 h (**B**), and 12 h (**C**). Each point represents the mean ± SD (*n* = 6). * *p* < 0.05 compared to control bLf.

Caco-2 cells were exposed to bLf and bLf-loaded SLPs (12.5–300 µg/mL) for different times. As illustrated in Figure 5A,B, the cells maintained over 90% viability after exposure to different concentrations of bLf and bLf-loaded SLPs for 4 and 8 h, respectively. This observation suggests that the formulations exhibited minimal toxicity, with cell viability exceeding 80%. After 12 h, bLf-loaded SLPs showed a higher inhibition effect on Caco-2 cells (* *p* < 0.05) compared with free bLf, especially when tested at a high concentration (Figure 5C). Table 2 presents the IC_50_ values after 4, 8, and 12 h of incubation with bLf, bLf-loaded unmodified, chitosan-, and pectin-modified SLPs, respectively. The IC_50_ values of each sample decreased as the incubation time increased. Based on the MTT results, the drug concentration of 100 µg/mL was selected for use for the uptake and transport studies (incubation time < 6 h).

### 3.4. In Vitro Qualitative and Quantitative Uptake of bLf and bLf-Loaded SLPs

**Figure 6:**
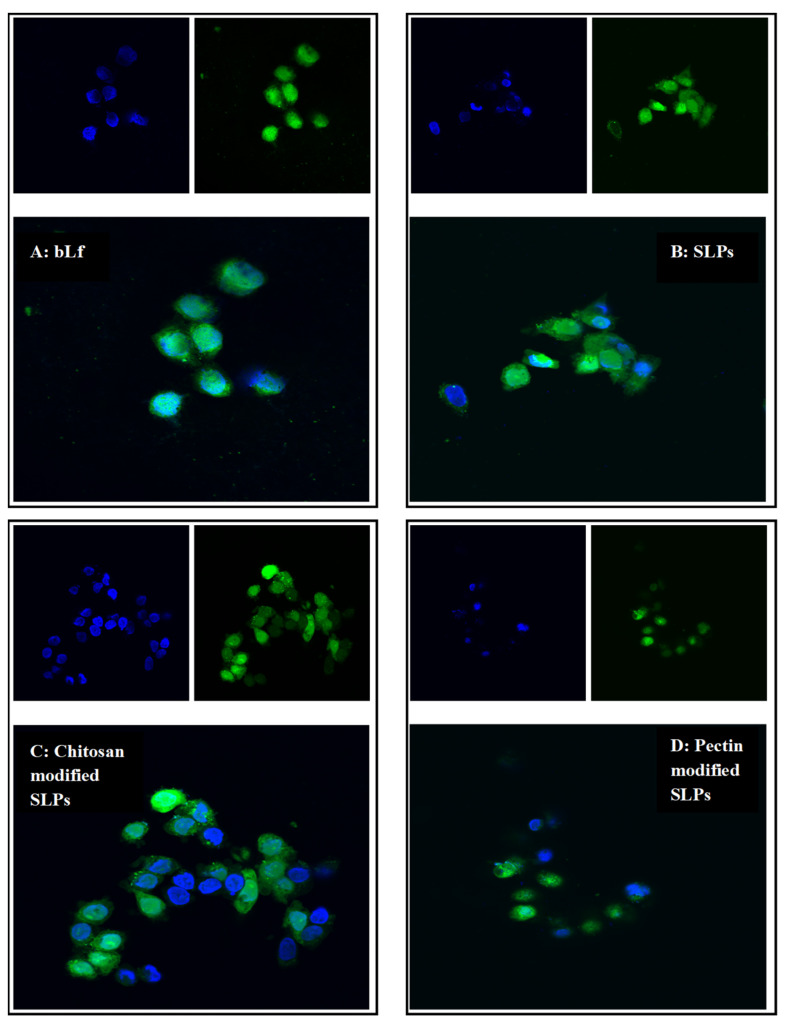
Confocal microscopy images of Caco-2 cells treated with FITC-bLf (**A**), FITC-bLf-loaded unmodified (**B**), chitosan- (**C**), and pectin-modified (**D**) SLPs for 1 h at 37 °C, respectively. Cell nuclei are stained blue with DAPI, FITC-bLf is shown as green fluorescence, and overlapped images are presented. Magnification = 600×.

The uptake of the FITC-bLf and FITC-bLf-loaded SLPs by Caco-2 cells at the same concentration of bLf (100 µg/mL) after an incubation time of 1 h was visualized using a confocal laser scanning microscope. Green fluorescence was detected in control cells exposed to the non-encapsulated FITC-bLf, showing that bLf was able to enter the cells (Figure 6A). Among the three types of FITC-bLf-loaded SLPs, all particles (visualized as green spots) were situated in close proximity to the cell nuclei (depicted in blue). This positioning suggests that the nanoparticles were successfully taken up by the cells (Figure 6B–D).

**Figure 7:**
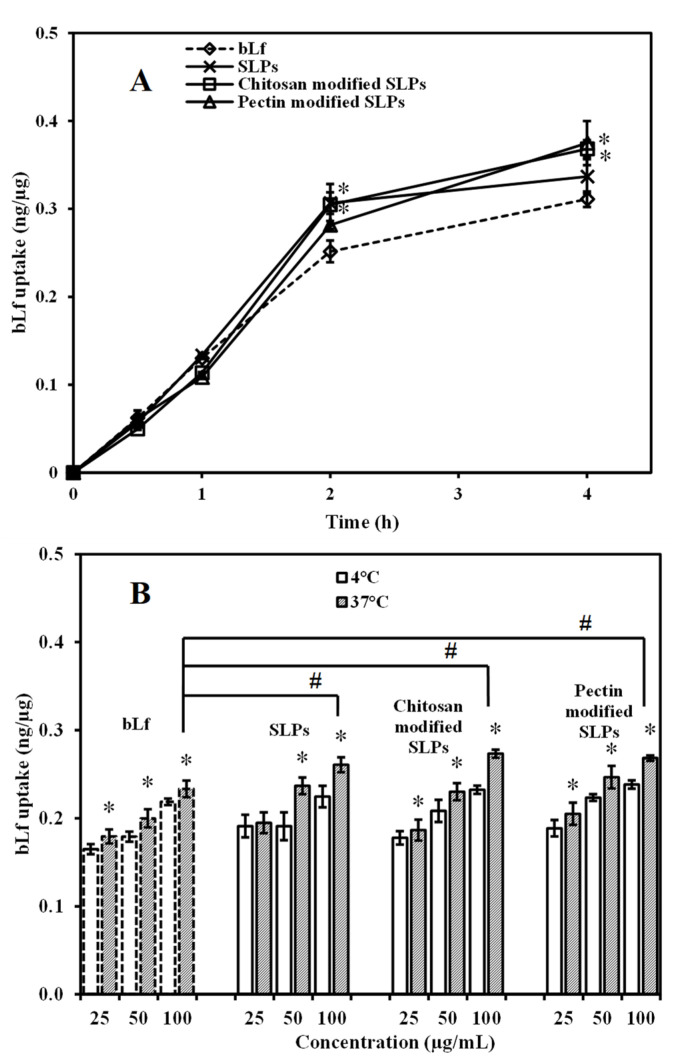
Cellular uptake of bLf, bLf-loaded unmodified, chitosan-modified, and pectin-modified SLPs by Caco-2 cells in HBSS buffer was examined. (**A**) The uptake over time of different drugs at a concentration of 100 μg/mL was observed at intervals of 0, 0.5, 1, 2, and 4 h at 37 °C. Statistical significance (* *p* < 0.05) was found in comparison to the control bLf. (**B**) The uptake after 4 h at both 4 °C and 37 °C was influenced by the drug concentration and temperature. Concentrations of 25, 50, and 100 μg/mL of various drugs were studied. Statistically significant differences (* *p* < 0.05) were identified when comparing to 4 °C, and (# *p* < 0.05) when comparing to the control bLf. Each data point represents the mean value ± standard deviation (SD) (*n* = 6).

The cellular uptake was further quantitatively investigated by ELISA and the BCA assay. The outcomes of the in vitro uptake assessment (depicted in Figure 7) revealed that the uptake of both bLf and bLf-loaded SLPs by Caco-2 cells followed patterns dependent on the time, temperature, and concentration. Specifically, the uptake of various formulations at a concentration of 100 µg/mL and at 37 °C exhibited an elevation in relation to the prolonged incubation duration (Figure 7A). The uptake values of encapsulated bLf were 0.34 ± 0.02 ng/µg for SLPs, 0.37 ± 0.01 ng/µg for chitosan-modified SLPs, and 0.38 ± 0.03 ng/µg for pectin-modified SLPs, which yielded a slight increase compared to those of the free bLf (0.31 ± 0.01 ng/µg) during 4 h of incubation (* *p* < 0.05). Furthermore, the quantities of bLf and bLf-loaded SLPs taken up by the cells were notably greater at 37 °C compared to 4 °C, indicating a reliance on energy for the uptake process. Across both temperature conditions of 4 °C and 37 °C, the uptake also exhibited a proportional increase with the rising concentrations when the cells were exposed to bLf and bLf-loaded SLPs within the concentration span of 25 to 100 μg/mL. Similarly, the uptake values of encapsulated bLf appeared to be higher than those of the free bLf at the concentration of 100 µg/mL under 4 h of incubation at 37 °C (* *p* < 0.05).

The presented experiments characterized and determined the effect of modified SLPs on the cellular uptake and transcellular transport of encapsulated bLf, aiming at providing basic information for the rational design of optimal drug carriers. Stearic acid, which is a type of long-chain fatty acid, was employed as the lipid component in our formulations. This particular fatty acid has been widely utilized in the creation of SLPs for different drugs and bioactive substances [25,44,45,46]. The choice of stearic acid as the lipid constituent was further supported by its recognition as safe by the United States Food and Drug Administration (FDA GRAS) for use as a food additive. This designation signifies its minimal to no toxicity for customers [44]. In addition, polymer modification using chitosan and pectin, which are also FDA GRAS materials, as natural polysaccharides, has been used in the development of various pharmaceutical formulations [47,48]. Although the formulation ingredients were carefully selected based on their biocompatibility, SLPs exerted some potential cytotoxic effects on Caco-2 cells at 12 h of incubation in our study. Cell viability in all cases was less than 80% of control cells. Opanasopit and colleagues also identified certain forms of chitosan that exhibited toxic effects on Caco-2 cells using the MTT assay [49]. Another possible reason could be the surfactant poloxamer 188, which had negative effects on cell viability. However, Fisher et al. documented that concentrations of up to 50 mg/mL of poloxamer 188 did not have any detrimental impact on cell viability or the integrity of the cell monolayer [50].

In this study, a FITC-labeled bLf signal was clearly observed in the confocal images, in which bLf was bound to the cell surface and internalized to the cells. The ELISA assay further confirmed the existence of bLf inside the cells via uptake. Comparable findings were illustrated in the study by Ashida and colleagues, where fluorescence-labeled bLf was shown to be taken up from the apical side and subsequently localized within the nuclei using human intestinal Caco-2 cells [51]. The precise cellular-level molecular mechanisms underlying the transportation of bLf remain largely uncharted. Receptor-mediated endocytosis/transcytosis was considered as the main mechanism of uptake of bLf by Caco-2 cells [52]. Among the proposed lactoferrin receptors are the low-density lipoprotein receptor-related proteins (LRPs) 1 and 2 [53,54,55], which belong to the LRP family of endocytic receptors [56,57]. Available evidence indicates that LRP1 serves a dual role as both an endocytic receptor and a signaling receptor [58,59,60,61]. With respect to the particles incorporating FITC-labeled bLf, the cellular uptake and transport appeared to be temperature- and energy-dependent, and the P_app_ increased when the P-gp inhibitor (verapamil) was added, which prompted us to speculate that the cellular uptake of SLPs primarily occurs through endocytosis, with active transportation facilitated by P-glycoprotein (P-gp) also playing a significant role [62,63,64].

### 3.5. Transport of bLf and bLf-Loaded SLPs

**Figure 8:**
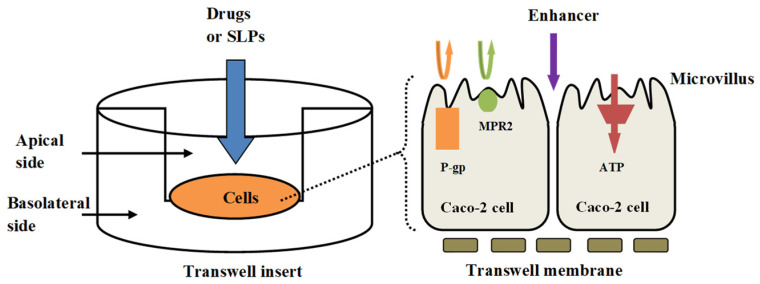
Illustration depicting transcellular transport and various transporters within Caco-2 cells.

**Figure 9:**
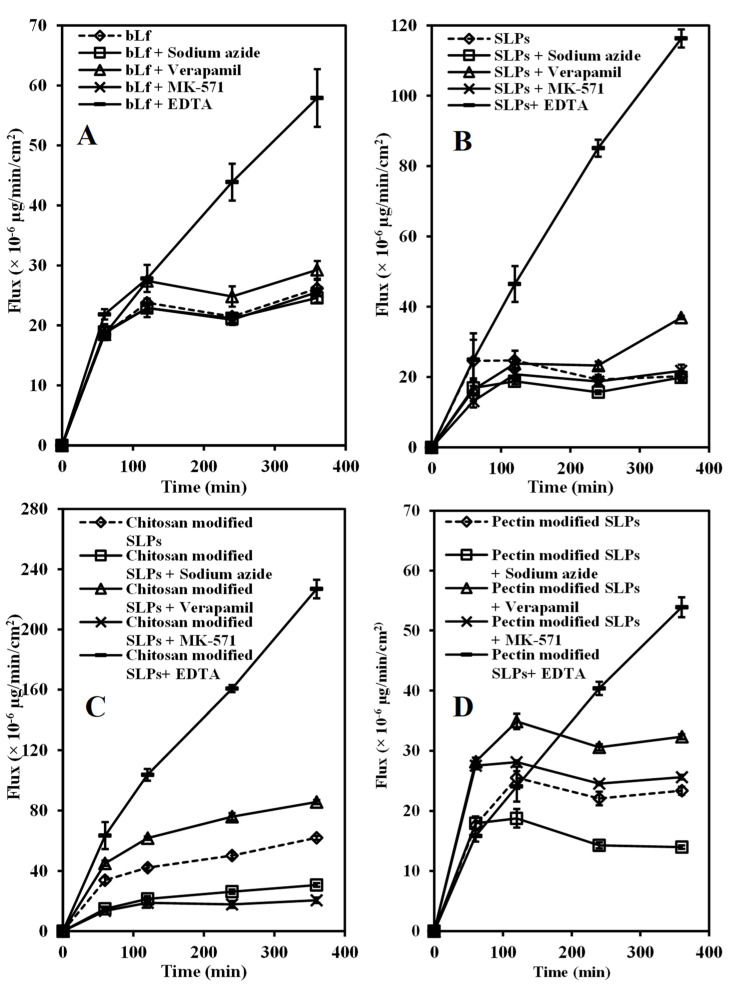
The impact of the ATP inhibitor (sodium azide), P-gp inhibitor (verapamil), MRP2 inhibitor (MK-571), and absorption enhancer (EDTA) on the transport rate of 100 μg/mL of bLf (**A**), bLf-loaded unmodified (**B**), chitosan-modified (**C**), and pectin-modified (**D**) SLPs over a span of 6 h at 37 °C is illustrated. Each data point corresponds to the mean value ± standard deviation (SD) (*n* = 6).

The intestinal transport mechanisms of bLf and bLf-loaded SLPs were investigated by adding various absorption enhancers or inhibitors. Figure 8 illustrates a schematic representation of transcellular transport, along with distinct transporters, within Caco-2 cells. The influence of absorption enhancers or inhibitors on the transport rate (the flux) of bLf and bLf-loaded SLPs is shown in Figure 9. For the control group, bLf, SLPs, and pectin-modified SLPs displayed similar transport patterns, with the maximum flux (~24 × 10^−6^ µg/min/cm^2^) observed at 2 h of incubation. After that, the transport rate tended to balance. In contrast, chitosan-modified SLPs exhibited the fastest flux, which gradually increased within 6 h, possibly due to the permeation enhancement effect of chitosan. The P_app_ values of all groups at 6 h are summarized in Table 3. Upon introduction of the ATP inhibitor (sodium azide at 10 mM), the flux kinetics of all groups consistently exhibited lower values, in contrast to the condition without sodium azide. The reduction was approximately 50% for chitosan-modified SLPs and around 40% for pectin-modified SLPs. These findings strongly suggest that the flux across the Caco-2 monolayer depends on energy, indicating the involvement of an active transport mechanism.

The introduction of the P-gp inhibitor (verapamil at 100 μM) led to a notable enhancement in the transport of bLf (approximately 12%), SLPs (around 83%), chitosan-modified SLPs (roughly 38%), and pectin-modified SLPs (about 39%). Conversely, the presence of the MRP2 inhibitor (MK-571 at 100 μM) did not exhibit any discernible increase. This observation suggests that P-gp is likely the primary contributor to the efflux transport of bLf across the Caco-2 cell membrane. Here, 10 mM of EDTA was used as a permeability enhancer. The flux of all formulations in the presence of EDTA basically linearly increased with time (Figure 9) and caused a significant increase in the permeability of all formulations compared with the control (* *p* < 0.05), with an increase of 1.2-fold to 4.75-fold.

In comparison to free bLf, the SLP formulations notably improved the absorption of the drug. However, the amount of bLf taken up from SLPs showed extremely low levels (only up to 0.38 ng taken up per µg of cell protein). This observation might be attributed to the limitations of using a single Caco-2 cell model and the unfavorable particle size of the particles. The characteristics of Caco-2 cells may not fully mirror the intricate physiology of the intestine, leading to inadequate correlations between in vitro and in vivo scenarios [65,66]. A more advanced in vitro intestinal permeability model involving a triple-cell-culture system was developed. This system incorporates enterocytes, mucus-secreting HT29-MTX cells, and M cells [67,68]. M cells exhibit a notable capacity for transcytosis and have the ability to transport a diverse array of materials, encompassing nanoparticles as well [69,70]. A study carried out by Yuan et al. [71] reported that up to 78% of the absorbed SLPs were conveyed into the systemic circulation through the lymphatic system, predominantly via the uptake by M cells. This pathway stands as the primary means of transport.

The increased uptake quantities in comparison to single Caco-2 cell monolayers can be attributed to the presence of M cells, which are likely to have a significant role in facilitating the entry of nanoparticles into the intestinal epithelium [72,73]. In more recent studies, the in vitro exploration of nanoparticles that have been modified on their surface and possess an average particle size smaller than 200 nm demonstrated improved efficiency in terms of cellular uptake within Caco-2 cells [74]. In the context of this research, the bLf-loaded SLPs that were formulated with a larger average particle size exceeding 200 nm could potentially be another contributing factor leading to reduced uptake and transport of these particles. On the other hand, Akiyama et al. [75] investigated the uptake of bLf using Caco-2 cells, suggesting that a major part of bLf molecules endocytosed by the cells are eventually degraded within the cell shortly after the localization to the early endosome, while a small part of the internalized bLf molecules escape from lysosomal degradation. All these factors contributed to low cellular uptake and resulted in low cellular transport (flux and P_app_) of SLPs. After confirming the impact of transporters in this investigation, it can be inferred that the endocytosis of SLPs might be influenced in part by apical proteins. Additionally, SLPs that have undergone endocytosis might encounter facilitated removal from the apical membrane through exocytosis, subsequently leading to a further reduction in transport efficiency. As a result, the P_app_ values of either bLf or bLf-loaded SLPs were less than 1 × 10^−6^, which were classified as poor-permeability compounds [76].

P-gp and MRP2 were transporters primarily expressed on the apical membranes of the epithelia. They performed vital functions in countering the absorption of drugs by the intestinal mucosa, achieving this by facilitating the efflux of drugs from the epithelial cells lining the intestine back into the intestinal lumen. This mechanism serves to prevent the drugs from being absorbed into the bloodstream [77,78]. Therefore, deactivation of the efflux pump may enhance the transport of drugs into the cells. Compared to the MRP2 inhibitor, inhibition of P-gp-mediated bLf elimination showed a major mechanism leading to an elevated bLf concentration in our study. These findings, along with studies by others, identity that P-gp plays the dominant role in restricting various drug plasma concentrations [79,80]. P_app_ from the AP to BL of all groups across the Caco-2 monolayer was significantly increased in the presence of chitosan or EDTA. Such effect is generally assumed to act on tight junctions between adjacent epithelial cells by manipulating the paracellular permeability. However, charged molecules or drugs with a molecular weight exceeding 400 to 600 Da are unable to traverse tight junctions, as the intercellular space within these junctions has a restricted dimension of around 7 nm, varying according to the specific cell type [81,82]. In particular, the permeability of 80 kDa bLf was more restricted by tight junctions. Therefore, numerous research endeavors have been centered on examining absorption enhancers that encompass compounds functioning through one or more mechanisms, in addition to the mere opening of tight junctions [83,84]. The mechanism of action to enhance the absorption of lipid particles could possibly be explained by reducing the extracellular calcium concentration (by adding EDTA) or decreasing the TEER of cell monolayers (due to the presence of chitosan), leading to a diminished viscosity of Caco-2 cells, increased fluidity of cell membranes, leakage of proteins through the membrane, and an enhanced endocytosis pathway [85,86,87,88].

The findings obtained from the Caco-2 cell experiments should not be immediately extrapolated to the in vivo context, as the impact observed would only transpire through direct contact with these substances and the mucosal surface. This type of interaction, which can be easily replicated in Caco-2 cells, might not fully mimic the complexity of the in vivo scenario. Nevertheless, the transport of nanoparticles could be significantly increased by using transport inhibitor or absorption enhancers and could be decreased in the presence of ATP inhibitors. This aligns with a recent significant study that comprehensively investigated the entire transportation process (including endocytosis, intracellular trafficking, exocytosis, and transcytosis) of nanoparticles within Caco-2 cells. The study highlighted that nanoparticles predominantly followed the endocytosis pathway.

## 4. Conclusions

Over the course of the 8 h investigation, SLPs exhibited time-dependent biocompatibility with Caco-2 cells, as evidenced by their lack of impact on cell viability and the absence of induction of apoptosis, as determined by the MTT assay. bLf can be internalized to cells via receptor-mediated endocytosis, while SLPs’ cellular internalization occurs via endocytosis as the dominating process and active transportation mediated by P-gp. The uptake of SLPs was time-, energy-, and concentration-dependent, which could remarkably enhance the absorption of bLf. The inhibitor of P-gp and the absorption enhancers significantly improved the cellular uptake of bLf or SLPs by using the Caco-2 cells. In future studies, SLPs will be employed as a potential carrier for oral delivery of macromolecular drugs.

## Figures and Tables

**Table 1:** The characteristics of bLf-loaded unmodified, chitosan-, and pectin-modified SLPs.

Delivery Systems	Particle Size (nm)	Polydispersity Index (Pdi)	Zeta Potential (mV)
SLPs	283.1 ± 4.4	0.41 ± 0.1	−12.43 ± 0.6
Chitosan-modified SLPs	518.5 ± 18.9	0.53 ± 0.1	−8.07 ± 0.4
Pectin-modified SLPs	459.5 ± 22.2	0.54 ± 0.0	−5.62 ± 0.3

**Table 2:** IC_50_ values of bLf, bLf-loaded unmodified, chitosan-, and pectin-modified SLPs on Caco-2 cells.

Incubation Time (h)	IC_50_			
	bLf	SLPs	Chitosan-Modified SLPs	Pectin-Modified SLPs
4	3026	2647	2524	2598
8	1152	1172	1010	1071
12	550	528	401.1	522.3

**Table 3:** The apparent permeability coefficients (P_app_ values) of bLf, bLf-loaded unmodified, chitosan-modified, and pectin-modified SLPs (at a concentration of 100 μg/mL) were determined as they moved from the apical to the basolateral chamber, following a 6 h exposure to various absorption inhibitors or enhancers. Statistical significance (* *p* < 0.05) was observed in comparison to the control.

	P_app_ (10^−9^ cm/s)				
	Control	Sodium Azide (10 mM)	Verapamil (100 µM)	MK-571 (100 µM)	EDTA (10 mM)
bLf	4.36 ± 0.2	4.09 ± 0.1 *	4.88 ± 0.3	4.26 ± 0.1	9.65 ± 0.8 *
SLPs	3.37 ± 0.3	3.31 ± 0.2	6.16 ± 0.1 *	3.63 ± 0.3	19.39 ± 0.4 *
Chitosan-modified SLPs	10.33 ± 0.2	5.11 ± 0.2 *	14.27 ± 0.2 *	3.42 ± 0.3 *	37.81 ± 1.0 *
Pectin-modified SLPs	3.90 ± 0.1	2.33 ± 0.1 *	5.39 ± 0.1 *	4.27 ± 0.1	8.98 ± 0.3 *

## Data Availability

Data is contained within the article.
